# Supplementary material for: OpenLabCluster: active learning based clustering and classification of animal behaviors based on kinematic body keypoints
Source: Front Syst Neurosci. 2025 Oct 31;19:1630654. doi: 10.3389/fnsys.2025.1630654 (PMC12615460; doi:10.3389/fnsys.2025.1630654)
Supplement: Supplementary file 1 [file Supplementary_file_1.pdf]

Supplementary Materials to OpenLabCluster: Active Learning Based Clustering and Classification of Animal Behaviors Based on Kinematic Body Keypoints

Jingyuan Li[1] Moishe Keselman[2] Eli Shlizerman[1,2]

[1]Department of Electrical and Computer Engineering,
[2]Department of Applied Mathematics,
University of Washington, Seattle, WA

1 Supplementary Methods

The inputs of OpenLabCluster are multi-dimensional time series representing the coordinates of animal body keypoints or other kinematic features tracked during movement along with video segments frames from which these were extracted. We denote the times-series as $\mathcal{X} = \{X_u \bigcup X_l\}$, with X_u representing the sequences in the unlabeled set and X_l in the labeled set, and assume that the segments are unlabeled, $\mathcal{X} = X_u$, in the beginning. A segment $\mathbf{x}_i \in \mathcal{X}$ is represented as a sequence $\mathbf{x}_i = [x_1,\ x_2,\ ..,\ x_t,\ ...,\ x_T]$, where x_t is the vector of features for movement at time t, $x_t \in R^p$. For the examples we consider here, the number of features is 16, 98, 16, 39 for Home-Cage Mouse, *C. elegans*, Zebrafish and Monkey datasets, respectively. OpenLabCluster includes three main components: (i) An encoder-decoder network that learns to reconstruct sequences and forms Latent Representations. (ii) A classifier network which performs behavior classification. (iii) AL selection which integrates Latent Representation information with the classifier output to optimize classification and annotation.

(i) Encoder-Decoder: We adopt the Predict & Cluster encoder-decoder framework which has been shown to achieve self-organizing latent representations [1, 2]. The encoder uses bidirectional Gated Recurrent Units (GRU) and receives $\mathbf{x_i} \in \mathcal{X}$ as input. The vector $\mathbf{h}_i^T$ is the latent representation which is the hidden state of the encoder GRU at the last time step T. It encodes the dynamic properties of the whole sequence $\mathbf{x_i}$ and lies in the latent space V, where $V = \{\mathbf{h}_i^T | \mathbf{h}_i^T = encoder(\mathbf{x_i}), \mathbf{x_i} \in \mathcal{X}\}$, i.e., the space spanned by the latent codes of all sequences. The unidirectional GRU-based decoder receives $\mathbf{h_i^T}$ and generates $\hat{\mathbf{x}}_i$ - the reconstruction of the original input sequences. The encoder-decoder network is trained by minimizing the reconstruction loss

$$\mathcal{L}_{re} = |\hat{\mathbf{x}}_i - \mathbf{x}_i|. \tag{1}$$

(ii) Classification: The classifier is a one-layer fully connected network appended to the encoder. It takes Latent Representation as input and generates the probabilities that a sample belongs to each behavioral states. During training, the classifier is learned to maximize the probability of the annotated behavior state. In other words, with the annotated samples given the classifier output, the classification loss is computed as

$$\mathcal{L}_{cla}^i = \sum_{l=1}^{C} -y_i^l \log(p^l(\mathbf{x}_i)), \tag{2}$$

where $y_i^l = 1$ if $\mathbf{x}_i$ belongs to class l, and $y_i^l = 0$ otherwise. The complete loss for each sample $\mathbf{x}_i$, is then composed from the reconstruction loss $\mathcal{L}_{re}^i$ and the classification loss $\mathcal{L}_{cla}^i$ for the annotated samples.

$$\mathcal{L} = \sum_{\mathbf{x}_i \in \mathcal{X}_l} \mathcal{L}_{cla}^i + \frac{1}{|\mathcal{X}|} \sum_{\mathbf{x}_i \in \mathcal{X}} \mathcal{L}_{re}^i, \tag{3}$$

where $|\mathcal{X}|$ is the total number of samples in the dataset. This includes all labeled samples annotated in current and earlier iteration.

(iii) AL: There are three AL methods embedded in OpenLabCluster: Cluster Center (Top), Uncertainty with Marginal Index (MI), Core-Set (CS).

Cluster Center (Top) leverages clusters information in the latent space to enhance coverage and effectiveness of selected segments (samples). Specifically, *K-Means* clustering is used to transform the latent representation into a collection of clusters $\mathcal{K}$. The number of clusters k

$$k = \frac{1}{N_{iter}} \times \ percentage \ \times |\mathcal{X}|, \tag{4}$$

is chosen based on the total number of selection iterations N_{iter} and the *percentage* of data would be annotated in total. k is fixed across selection iterations such that k is the number of samples to be annotated and each is located in a different cluster.

Marginal Index (MI) is based on the classifier output and measures the difference between the classifier output, evaluating top two difference of p. The probability prediction p of each class l is

$$p^l(\mathbf{x_i}) = p^l(\hat{y}_i = l | W_\theta, W_\delta) = \mathcal{C}^l(\mathbf{x_i}),$$

where p^l denotes the probability of a sample to belong to a class l among C classes ($l \in [1, C]$) predicted by the classifier $\mathcal{C}$. $\mathcal{C}$ indicates the transformation with the classifier. MI is computed as the measure of the confidence, difference between the most probable class and the second most probable class [3, 4], i.e.,

$$MI = \max_{l \in [1:C]} \left(p^l\right) - \max_{l \in ([1:C] \setminus l^*)} \left(p^l\right), \tag{5}$$

where $l^* = \arg\max_{l \in [1:C]} \left(p^l\right)$.

Core-Set (CS) aims to discover a set of samples that can cover unlabeled samples with a certain radius [5]. The algorithm finds a set of samples such that the radius is minimal.

With the labeled samples, the classifier is trained until the classification accuracy on these labeled samples converges. Annotation iteration repeats until reaching the annotation budget.

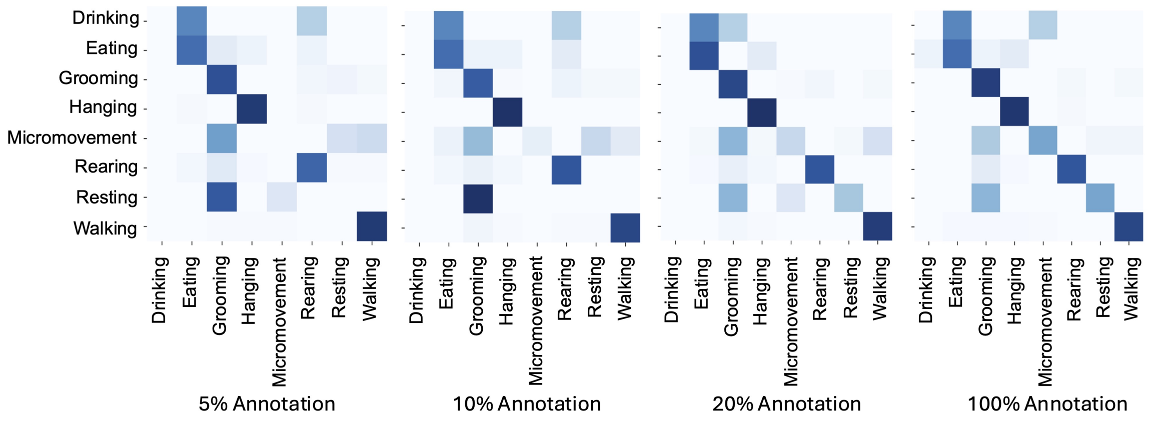

Figure 1: Visualization of confusion matrix on Home-Cage Mouse dataset under different annotation level (5%, 10%, 20%, 100%).

1.1 Home-Cage Mouse Behavior Analysis

To provide the detailed behavior classification results. We show confusion matrices on Home-Cage Mouse dataset obtained with OpenLabCluster-V CS across different annotation budget (5%, 10%, 20%, 100%) in Figure 1.

1.2 Zebrafish Behaviors and Dynamics

Zebrafish behavior is subdivided into 13 classes: approach swims (ASs), slow types 1 (S1), slow types 2 (S2), short and long capture swim (SCS and LCS), burst type forward swim (BS), J-turns, high-angle

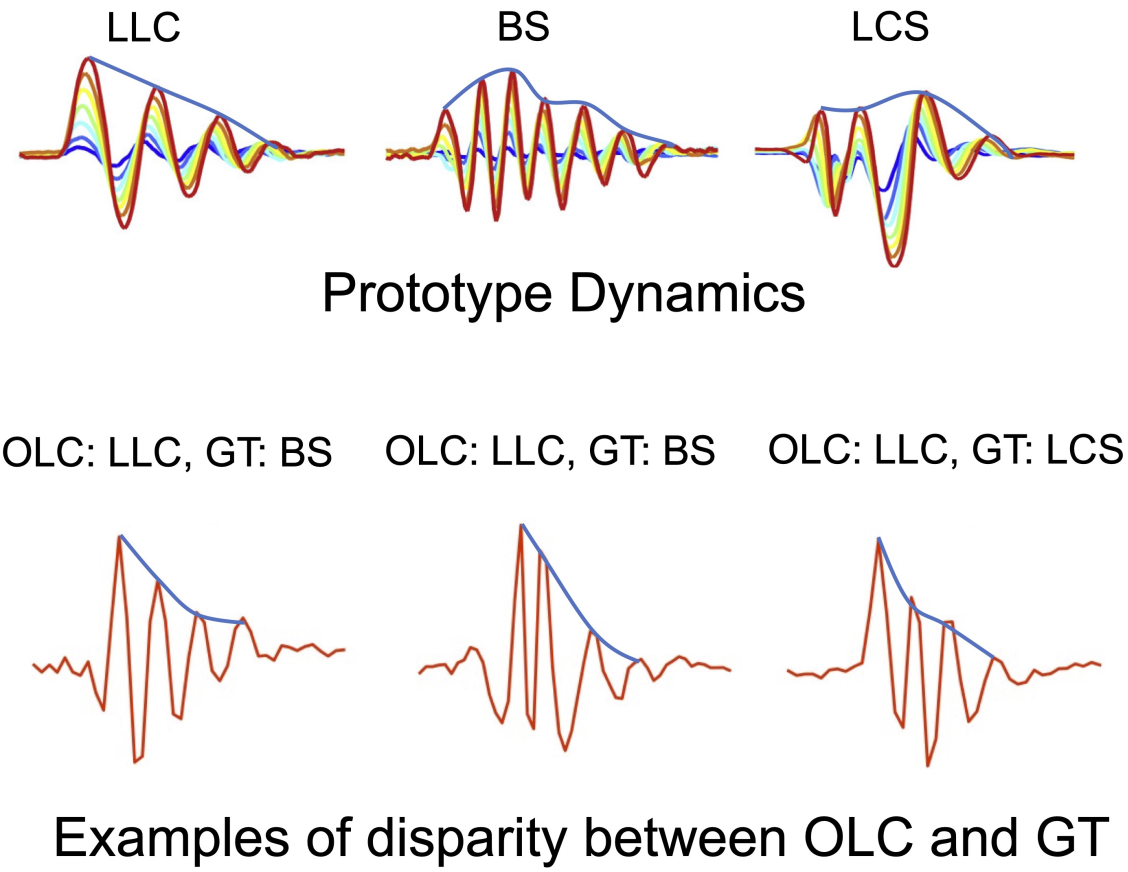

Figure 2: Top: Prototypical dynamics of three behavioral states (LLC, BS, LCS), envelop (blue). Bottom: Three examples of disparity between OpenLabCluster and Ground Truth (GT) classification.

turn (HAT), C-start escape swims (SLC), long latency C-starts (LLC), O-bends, and routine turns (RTs), spot avoidance turn (SAT).

As shown in Fig **??**C, OpenLabCluster could “misclassify" samples to belong to LLC class, although they have been annotated by previous analysis as BS or LCS. Since the annotation is obtained by a prior unsupervised learning method and not a case-by-case manual annotation, it could be possible that the annotation is made incorrectly, especially for those segments that are located near the boundaries of clusters. We show several of such conflicting examples in Fig. 2. In the top row, we show the typical dynamics of three ground-truth (GT) classes (BS, LCS, and LLC). Inspecting the prototypical dynamics of the LCS, BS, and LLC, one can find that each profile has unique dynamic profile. For example, LLC profile peaks in the beginning and gradually decays. In the bottom row of Fig. 2, we show three examples of segments classified by OpenLabCluster as *LLC* but marked differently by prior annotation that we consider as ground-truth. It appears that while these examples are similar to LLC profile (having a peak in the beginning and decay) supporting the decision of OpenLabCluster to classify these segments as LLC, the ground truth annotations made by prior clustering analysis are contradictory.

2 Supplementary Ablation study

The two components of OpenLabCluster of unsupervised clustering and semi-supervised classification are essential to the accuracy. In Table 1 we examined “ablated" versions of OpenLabCluster to demonstrate the impact of each component; *C* designates a variant that includes a classifier network only. *RC* designates a variant where pretraining phase of OpenLabCluster is ablated, i.e., all the weights of the encoder-decoder and the classifier are randomly initialized. In this scenario, there is no pre-organized Latent Representation. *IRC* designates a variant where AL is not being applied but the encoder-decoder

	Mouse			Zebrafish			*C elegans*		
Label(%)	5	10	20	5	10	20	5	10	20
Label(#)	143	286	571	265	530	1059	27	55	109
C	55.2	54.1	64.5	72.5	70	75.8	71.3	75.2	77.5
RC	53.7	54.1	63.9	66.3	72.3	74.8	71.0	76.4	79.1
IRC	63.0	66.6	76.1	72.8	75.9	79.0	73.8	**77.0**	77.3
OpenLabCluster CS	**65.3**	75.4	**82.2**	71.9	76.6	79.6	73.5	64.3	75.4
OpenLabCluster Top	58.5	58.4	76.4	72.0	77.1	80.1	76.5	76.5	**77.8**
OpenLabCluster MI	63.4	**76.3**	78.9	**74.2**	**79.1**	**81.1**	**76.7**	76.8	77.0

Table 1: Comparison of OpenLabCluster against its ablated versions: RC, IRC for 5%, 10% and 20% annotations on Mouse , Zebrafish and *C. elegans* datasets.

structure as well as the training paradigm including pretraining is kept the same as in OpenLabCluster (with pre-organized Latent Representation). As shown in Table 1, the accuracy of IRC is higher than that of C and RC showing the importance of pre-organized encoder-decoder Latent Representation. OpenLabCluster along with various AL approaches further enhances the accuracy (over IRC) in most cases, especially on the Home-Cage Mouse and the Zebrafish datasets with an average improvement 5.78%. The reason that improvement of OpenLabCluster is not significant on the *C. elegans* dataset could be that the diversity is limited on the dataset, i.e., samples are equally informative for behavior states learning.
